# The Gestational Obesity Weight Management: Implementation of National Guidelines (GLOWING) study: a pilot cluster randomised controlled trial

**DOI:** 10.1186/s40814-024-01450-2

**Published:** 2024-03-01

**Authors:** Nicola Heslehurst, Catherine McParlin, Falko F. Sniehotta, Judith Rankin, Lisa Crowe, Elaine McColl

**Affiliations:** 1 Population Health Sciences Institute, Newcastle University, Baddiley Clark Building, Newcastle Upon Tyne, NE2 4AX UK; 2 Faculty of Health and Life Sciences, Northumbria University, Newcastle Upon Tyne, UK

**Keywords:** Implementation, Guidelines, Behaviour change, Midwives, Pregnancy, Obesity, Weight management, Cluster RCT, Pilot study

## Abstract

**Background:**

Pregnancy weight management interventions can improve maternal diet, physical activity, gestational weight gain, and postnatal weight retention. UK guidelines were published in 2010 but health professionals report multiple complex barriers to practice. GLOWING used social cognitive theory to address evidence-based barriers to midwives’ implementation of weight management guidelines into routine practice. This paper reports the pilot trial outcomes relating to feasibility and acceptability of intervention delivery and trial procedures.

**Methods:**

GLOWING was a multi-centre parallel-group pilot cluster RCT comparing the delivery of a behaviour change intervention for midwives (delivered as training workshops) with usual practice. The clusters were four NHS Trusts in Northeast England, randomised to intervention or control arms. Blinding of allocation was not possible due to the nature of the intervention. We aimed to deliver the intervention to all eligible midwives in the intervention arm, in groups of 6 midwives per workshop, and to pilot questionnaire data collection for a future definitive trial. Intervention arm midwives’ acceptability of GLOWING content and delivery was assessed using a mixed methods questionnaire, and pregnant women’s acceptability of trial procedures by interviews. Quantitative data were analysed descriptively and qualitative data thematically.

**Results:**

In intervention arm Trusts, 100% of eligible midwives (*n* = 67) were recruited to, and received, the intervention; however, not all workshops had the planned number of attendees (range 3–8). The consent rate amongst midwives randomised (*n* = 100) to complete questionnaires was 74% (*n* = 74) (95% CI 65%, 83%), and overall completion rate 89% (*n* = 66) (95% CI 82%, 96%). Follow-up response rate was 66% (*n* = 49) (95% CI 55%, 77%), with a marked difference between intervention (39%, *n* = 15) and control (94%, *n* = 34) groups potentially due to the volume of research activities. Overall, 64% (*n* = 47) (95% CI 53%, 75%) completed both baseline and follow-up questionnaires. Midwives viewed the intervention as acceptable and directly relevant to routine practice. The least popular components related to scripted role-plays. Pregnant women viewed the recruitment and trial processes to be acceptable.

**Conclusions:**

This rigorously conducted pilot study demonstrated feasibility intervention delivery and a high level of acceptability amongst participants. It has provided information required to refine the intervention and trial protocol, enhancing confidence that a definitive trial could be carried out.

**Trial registration:**

ISRCTN46869894; retrospectively registered 25th May 2016, www.isrctn.com/ISRCTN46869894.

**Supplementary Information:**

The online version contains supplementary material available at 10.1186/s40814-024-01450-2.

## Key messages regarding feasibility

- What uncertainties existed regarding feasibility?
  The main feasibility question was whether a 1-day training intervention, requiring midwives to be released from clinical practice for the duration, could be delivered to all eligible midwives
- What are the key feasibility findings?
  The intervention was delivered to 100% of eligible midwives, albeit with fewer midwives per session in the smaller NHS Trust due to clinical capacity of the team, and was acceptable to participants
- What are the implications of the feasibility findings for the design of the main study?
  Delivering the intervention to midwives from different Trusts combined could help overcome issues of clinical capacity. Burden of data collection needs to be reduced for a future trial.

## Background

In the UK, prevalence of maternal obesity has increased from 7.6% of all pregnancies in 1989 [1] to 22.1% in 2019 [2] disproportionally affecting women with low socio-economic status and minoritised ethnic groups [3]. Maternal obesity has short- and long-term implications for women and babies, including mortality, gestational diabetes, reduced breastfeeding, congenital anomalies and childhood obesity development [4–6]. However, pregnancy is a unique opportunity for weight management interventions as pregnant women are motivated and receptive to nutrition advice, and interventions have potential to impact on lifelong health of women and children [7–10]. Policy makers recognise the importance of health professionals providing support for weight gain, weight retention and long-term obesity development [11, 12]. While there is some inconsistency between individual trials, a systematic review of systematic reviews identified overall patterns for improvements in maternal diet and physical activity (PA) behaviours during pregnancy and beyond, including increased fruit and vegetable consumption, reduced sugar and saturated fat consumption, and reduced decline in moderate PA over the duration of pregnancy [13]. Similarly, interventions delivered during pregnancy consistently reduce gestational weight gain and postnatal weight retention, and some meta-analyses show significant reductions in caesarean delivery and gestational diabetes [3, 14–16]. Process evaluations of interventions identify the importance of frequent and personal interactions with health professionals [17, 18], supporting the need to embed weight management support into routine maternity care [19].

Weight management recommendations are included in the UK and international pregnancy guidelines [20, 21]. UK National Institute of Health and Care Excellence (NICE) guidelines include recommendations for health professional advice and support, including discussing obesity risks, weight-related behaviour, incorporating practical and tailored advice, and being sensitive to women’s weight concerns [22]. However, passive dissemination of guidelines is an ineffective means of implementation into clinical practice, reducing the chance of positive health outcomes compared with more active strategies [23, 24]. Midwives report a lack of knowledge, skills and confidence in their ability to provide support for women with obesity in pregnancy [25], resulting in inconsistent and ad hoc advice for pregnant women [1, 9, 26, 27]. They have expressed the need for support to help them to overcome barriers to practice, comparing obesity with other complex topics for which training is available [9, 25, 26]. Pregnant women also report receiving inadequate or conflicting information about nutrition and PA from health professionals [7, 28]. Health professional capacity-building is required to improve maternal nutrition and child health [29], and should include developing knowledge and skills to implement NICE guidelines [22, 30].

Pilot studies are required to inform the design and conduct of a definitive trial to reduce uncertainty and optimise the chances of a successful summative evaluation, especially when the intervention has multiple components [31–33]. This paper reports the results of the GLOWING pilot trial. The protocol is registered with ISRCTN (ISRCTN46869894) and published [34].

## Methods

This pilot study is reported using the CONSORT extension to randomised pilot and feasibility trials [35] (see Additional file 1).

### Aim

The aim of the GLOWING pilot, conducted between February 2016 and June 2019, was to test whether it would be feasible and acceptable to deliver a theory-based behaviour change intervention to support midwives’ implementation of weight management guidelines (see Additional file 2). The specific pilot study objectives reported here relate to assessing the feasibility and acceptability of intervention delivery and trial procedures, including data collection [34].

### Design and setting

The GLOWING external rehearsal pilot trial was a multi-centre parallel-group cluster RCT comparing the delivery of a theory-based behaviour change intervention for midwives with usual practice. The clusters were four NHS Trusts providing maternity care in Northeast England, UK: two provided services to a large population (3000–6000 births/year) and two were smaller (1000–2000 births/year). Computer randomisation of Trusts to intervention or control arms, stratified by cluster size, was carried out by a statistician using anonymised unique IDs to prevent allocation bias. Baseline data collection from midwives and pregnant women was carried out before cluster randomisation (i.e. allocation concealment before consent and data collection). For follow-up data collection, it was not possible for the intervention delivery team or midwives to be blinded. Pregnant women were not aware of their randomisation arm.

### Participants

Eligible participants were all community midwives, and hospital-based midwives with a specific or specialist role relating to maternal obesity, weight management or public health. In intervention sites, we aimed to deliver the intervention to all eligible midwives. To receive 30 returned pre- and post-intervention questionnaires from midwives per arm [32], we aimed to randomly select 50 midwives per arm: 30 from the larger Trusts, and 20 from the smaller Trusts (note: one participating NHS Trust had fewer than 20 eligible midwives, see Table 3; therefore, all were selected without randomisation). This randomisation assumed a data collection consent rate of 72% (i.e. to consent and send questionnaires to 36 midwives/arm; 18 per Trust), and retention rate of 83% (i.e. to receive returned questionnaires from 30 per arm; 15 per Trust). The questionnaires were developed for the GLOWING study and tailored to the guideline recommended behaviours; they are reported elsewhere [36]. They included sections on midwives’ self-reported weight communication and weight management practice and the social cognitive theory constructs of self-efficacy, outcome expectancies and intentions. We also asked midwives to complete the Beliefs About Obese Persons (BOAP) scale [37], and qualitative vignettes of simulated practice developed using evidence-based barriers to practice.

The intervention did not target pregnant women directly. However, pregnant women aged ≥ 18 years, with singleton pregnancies and pre-pregnancy obesity (BMI ≥ 30 kg/m^2^), were recruited to pilot data collection, and provided interview data for the process evaluation. Women were given a £10 voucher for every data collection episode: pre-intervention participants had one episode (at a routine ultrasound appointment), and a new group of participants were recruited post-intervention and these had up to six episodes (one at a routine ultrasound appointment, one-third trimester, and four postnatal up to 12 months). We aimed for an achieved sample of 30 women/arm [32] pre-intervention to test data collection procedures. Following intervention delivery, we aimed to recruit another 30 women/arm to provide pregnancy and postnatal outcome data, and seven women/cluster for semi-structured interviews. Allowing for attrition, we aimed to approach 50 women/arm for data collection in each phase (pre- and post-intervention) and to obtain consent from a minimum of 36 women/arm (18/Trust) for questionnaires. The questionnaire included women’s report of their midwives’ behaviours and were developed for this study (e.g. advice and support provided relating to weight management), a food frequency questionnaire (FFQ, originally adapted by the UPBEAT Trial team [38, 39]), the Pregnancy Physical Activity Questionnaire (PPAQ) [40] and a validated questionnaire exploring psychosocial measures for understanding weight-related behaviours in pregnancy [41]; these data are reported elsewhere [42]. We also explored the feasibility of measuring changes in women’s weight during pregnancy and postnatally. At the time of study, NICE guidelines did not recommend routine weighing of women unless clinically indicated [22], whereas obstetric guidelines recommended that women with obesity were weighed at delivery to inform the dosage required for thromboprophylaxis medication [30]. Therefore, we explored the availability of extracting routine weight measurements from medical records (pre- and post-intervention participants). Post-intervention, we additionally asked all participants to have their weight measured at each follow-up data collection time point (third trimester and postnatal). We provided cards to be completed and signed by a health professional, or women could return evidence of weight measurements (e.g. print out from pharmacy scales).

Inability to speak or read English was an exclusion criterion, as questionnaires were not validated in other languages. Written informed consent was obtained from midwives and pregnant women prior to any level of participation, with additional consent being requested and obtained for qualitative interviews. Questionnaires were distributed by the Reproductive Health and Childbirth Research Teams at each NHS Trust with pre-paid return envelopes addressed to the university research team. The Trust research teams sent up to three reminders to recruited midwives to complete their questionnaires. No reminders were sent to recruited women as their questionnaires were primarily asking them to reflect on their booking appointment discussions with their community midwife and there was potential for recall bias if there were subsequent appointments with their midwives which they may have reflected on (e.g. usual care is to have a 16-week antenatal appointment).

### Intervention development, content and delivery

The intervention development followed a four-step approach for developing theory-informed interventions to change clinical practice [43], described elsewhere [34] using the template for intervention description and replication (TIDieR) checklist and guide [44]. The intervention drew on social cognitive theory to address evidence-based barriers to health professional practice [34]. GLOWING comprised a full-day (6 h) of intensive face-to-face training for small groups of midwives (target of *n* = 6/day), plus the provision of training resources for their continued professional development, and a year’s supply of ‘GLOWING packs’ (information resources for midwives to share with pregnant women during routine practice). The training comprised five components: introduction, weight communication, weight management, consolidation of the day and summary and evaluation. These included a combination of didactic and interactive activities including lectures, videos, group discussion, reflection, scripted role-play activities and developing action plans (see Additional file 3). GLOWING was delivered in local NHS Trust settings, by the research lead (NH) and research midwife (CM). The facilitators had a script to standardise the content and delivery of each workshop. However, the interactive nature of the intervention required the facilitators to be responsive to midwives’ discussions and questions on the day. The first GLOWING workshop was used to pilot the timing of intervention delivery and refine the content to ensure all essential elements could be delivered. Participating midwives were provided with an evaluation form to assess their acceptability of the intervention and its relevance to practice (see Additional file 4). This included quantitative questions on each component of the intervention, the resources provided and the facilities and training delivery, with free text sections for midwives to provide additional feedback. Midwives were prompted to complete the form throughout the day after each component was delivered. Midwives working for the Trusts in the control arm did not receive any training or have any contact with the research team. They continued their usual practice.

### Outcomes and analysis

The main outcomes of the pilot study were feasibility and acceptability of the intervention and trial procedures (Table 1). This included the feasibility of delivering the intervention as planned; recruitment and randomisation of sites and participants; and collecting the outcome measures required for a definitive trial. The proposed primary and secondary outcomes for a future definitive trial were measures of midwifery practice in respect of weight management, pregnant women’s experience of receipt of advice; and measures of women’s weight and diet and physical activity behaviours.

**Table 1:** Pilot trial outcomes and methods of assessment

Outcome	Assessment
Feasibility of delivering the intervention as planned	a. Participation rate of midwives attending the intervention delivery (training day) in the intervention arm, calculated as a percentage of all eligible midwives invited to attend b. Feasibility of intervention delivery, calculated as the number of intervention sessions delivered with the planned number of midwives (six/session) in attendance at each session c. Intensity of intervention delivery, calculated as the number of intervention sessions required to deliver the intervention to all recruited midwives in the intervention arm d. Time required for intervention delivery, calculated for both intervention sites as the number of weeks between delivery of the first and last intervention session e. Fidelity of intervention delivery following the initial pilot intervention session, calculated as the frequency of the delivery of the intervention as planned (i.e. all content delivered in the allocated time) measured by direct observation and video recording of the intervention sessions, and frequency of deviation from protocol f Time taken to deliver intervention sessions, obtained from analysis of videos of each intervention delivery
Feasibility of recruitment and randomisation of sites, and recruitment of midwives and pregnant women	a. Rates of recruitment of sites b. Achievement of randomisation c. Rates of recruitment of midwives (to receive intervention and for data collection) and of pregnant women (for data collection)
Feasibility of collecting the outcome measures required for a definitive trial, and to prioritise which outcomes should be primary or secondary outcomes for a definitive trial	a. Rates of consent and attrition of midwives and pregnant women to provide questionnaire data at all time points: i Midwives’ questionnaires at baseline (pre-intervention) and follow-up (3 and 6 months post-intervention) ii Women’s questionnaires at baseline (pre-intervention at 20 weeks gestation) iii Women’s questionnaires and weight measurements at follow-up (post-intervention at 12 and 36 weeks gestation, and 3, 6, 9 and 12 months postnatal) b. Completeness of questionnaire data returned, calculated as percentage of missing data for each questionnaire section c. Feasibility of using routine hospital data for pregnancy weight measurements, assessed by a clinical audit of electronic and handheld medical records for all participating women at baseline and follow up

Descriptive statistical analysis was carried out for quantitative data, including percentages with 95% confidence intervals (CI), and means with standard deviations (SD). The qualitative interview data were analysed by a researcher not otherwise involved in GLOWING (LC) using a deductive thematic analysis approach to explore acceptability of trial procedures. The free text elements of the evaluation forms assessing acceptability and relevance of the GLOWING intervention amongst intervention arm midwives were also analysed using thematic analysis. The purpose of the descriptive and thematic analysis was to inform the development and refinement of the intervention and trial methods for a future definitive trial. We did not, however, set any explicit criteria, with respect to rates of eligibility, consent, randomisation, retention, etc., for continuation (with or without change) to a definitive trial.

## Results

### Feasibility of recruitment and randomisation

**Fig. 1:**
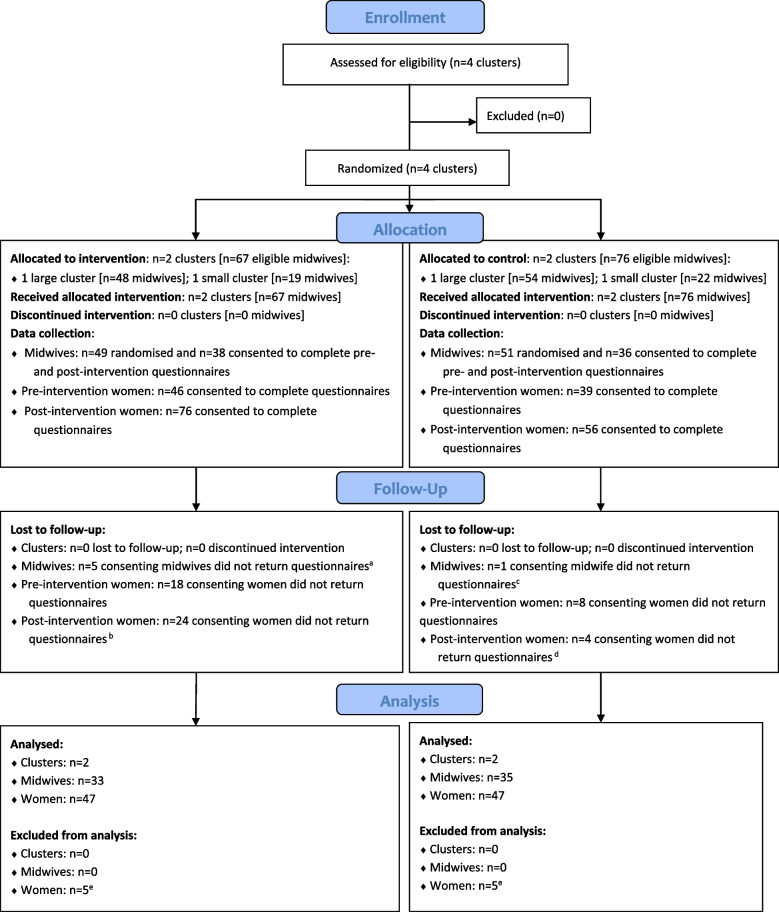
CONSORT flow chart. Legend: ^a^Intervention arm: *n* = 33 midwives returned ≥ 1 questionnaire. ^b^Intervention arm: *n* = 52 women returned ≥ 1 post-intervention questionnaire. ^c^Control arm: *n* = 35 midwives returned ≥ 1 questionnaire. ^d^Control arm: *n* = 52 women returned ≥ 1 post-intervention questionnaire. ^e^Excluded due to potential contamination between trial arms due to women being recruited at different sites to their booking appointment

Four NHS Trusts were approached to participate in GLOWING and all four agreed (Fig. 1). Cluster randomisation of the recruited Trusts was straightforward and completed following baseline data collection. Within each Trust, research midwives screened staff lists to identify all eligible midwives. In intervention arm Trusts, 100% (*n* = 67) of eligible midwives were recruited to, and received, the intervention (Table 2). Rates of recruitment and retention for questionnaire data collection are summarised in Table 3. In the two larger clusters (Trusts 1 and 3), just over 60% (*n* = 63: *n* = 30 Trust 1; *n* = 33 Trust 3) of midwives were randomised to receive questionnaires, whereas in the smaller trusts, 100% (*n* = 19) (Trust 2) and 82% (*n* = 18) (Trust 4) of eligible midwives were randomised and approached; the overall rate of randomisation was 70% (*n* = 100) (95% CI 62%, 77%). Consent rates amongst those randomised ranged from 55% (*n* = 18) to 100% (*n* = 18) with an overall consent rate of 74% (*n* = 74) (95% CI 65%, 83%), in line with expectations.

**Table 2:** Feasibility of delivering the intervention as planned

	**Large NHS Trust**	**Small NHS Trust**	**Total**
Eligible midwives (*n*)	48	19	67
Midwives recruited to GLOWING session (*n*, %)	48 (100%)	19 (100%)	67 (100%)
Midwives attended GLOWING session (*n*, %)^a^	48 (100%)	19 (100%)	67 (100%)
GLOWING sessions delivered (*n*)^b^	8	5	13
Midwives per session range (mean)	4–8 (6)	3–4 (4)	3–8 (5)
GLOWING delivered to 6 midwives/session as planned (*n*, %)^c^	3 (38%)	0 (0%)	3 (23%)
GLOWING delivered to fewer midwives/session than planned (*n*, %)	2 (25%)	5 (100%)	7 (53%)
GLOWING delivered to more midwives/session than planned (*n*, %)	3 (38%)	0 (0%)	3 (23%)
Time required for intervention delivery (weeks)^d^	14.3	7.0	14.3
Pilot delivery (*n*)^e^	1	0	1
Delivery of the intervention per protocol (*n*, %)^e^	7 (100%)	5 (100%)	12 (100%)

^a^Recruitment rate of midwives attending the intervention delivery (training day) in the intervention arm, calculated as a percentage of all eligible midwives invited to attend

^b^Intensity of intervention delivery calculated as the number of intervention sessions required to deliver the intervention to all recruited midwives in the intervention arm

^c^Feasibility of intervention delivery, calculated as the number of intervention sessions delivered with the planned number of midwives (six/session) in attendance at each session

^d^Time required for intervention delivery, calculated for both intervention sites as the number of weeks from delivering the first and last intervention session

^e^Fidelity of intervention delivery following the initial pilot intervention session, calculated as the frequency of the delivery of the intervention as planned (i.e. all content delivered in the allocated time) measured by direct observation and video recording of the intervention sessions, and frequency of deviation from protocol

**Table 3:** Recruitment and retention rates of midwives to questionnaire data collection

		Eligible	Randomised (denominator eligible)		Consented (denominator randomised)		Baseline returned (denominator consented)^a^		Follow-up return (denominator consented)^b^		Both returned (denominator consented)	
		*n*	*n*	%	*n*	%	*n*	%	*n*	%	*n*	%
**Intervention**	Trust 1 (large)	48	30	63%	20	67%	18	90%	9	45%	8	40%
	Trust 2 (small)	19	19	100%	18	95%	14	78%	6	33%	6	33%
	**Total**	**67**	**49**	**73%**	**38**	**78%**	**32**	**84%**	**15**	**39%**	**14**	**37%**
**Control**	Trust 3 (large)	54	33	61%	18	55%	16	89%	17	94%	16	89%
	Trust 4 (small)	22	18	82%	18	100%	18	100%	17	94%	17	94%
	**Total**	**76**	**51**	**67%**	**36**	**71%**	**34**	**94%**	**34**	**94%**	**33**	**92%**
**All**	**Overall total**	**143**	**100**	**70%**	**74**	**74%**	**66**	**89%**	**49**	**66%**	**47**	**64%**

^a^NHS Trusts 1, 2 and 3 each had one midwife whose questionnaire was reported to be lost in the post at baseline, the number in the table relates to the number we received back rather than the number of midwives who reported they had already sent it when we followed up non-responders

^b^We only collected follow-up date for midwives at one time point rather than two time points as per the protocol given the poor retention rate in the intervention arm and the need to prioritise exploring this further with non-responding midwives rather than burden them with further questionnaire data collection

The initially planned recruitment procedure for pregnant women to receive questionnaires was to randomly select eligible women to be approached by research midwives at their 20-week ultrasound scan at the hospitals (as described in the published protocol [34]). Data on this method of recruitment are presented in Table 4. Based on electronic records, a total of 596 women were deemed eligible for randomisation. It was, however, difficult to assess accurately all eligibility criteria (e.g. ability to speak English) from these records, so this is likely to be an over-estimate. Given the uncertainty of the number of women to approach for recruitment of 18 per Trust, three rounds of randomisation of (potentially) eligible women were conducted in an effort to identify 50 women per Trust to further screen handheld records for eligibility and approach for recruitment. In one of the small Trusts, even after three rounds, only 41 women could be randomised based on eligibility criteria. Rates of randomisation relative to eligibility ranged from 29 (*n* = 50) to 100% (*n* = 58), with an overall rate of 33% (199) (95% CI 30%, 37%). However, only 47% (51) (95% CI 40%, 54%) of those randomised across all clusters were actually approached to take part. Data on reasons for women not being approached were provided by three of the clusters (Trusts 1, 2 and 4; Trust 3 did not record reasons). Across the board, the most common issues preventing women being approached were related to scan appointments (43%). This included scan dates being changed, women not attending, no scan appointment being logged on the appointment system or the research team missing the woman in the scan clinic. The second most common explanation (main reason in Trust 1) related to lack of research midwife availability for all clinics (31%). Nine randomised women (13%) were not approached due to miscarriage or termination of pregnancy between randomisation and the 20-week ultrasound scan. A further seven (10%) were found to be ineligible for reasons not recorded on e-records. Trust 1 was unable to approach two women because they ran out of questionnaires. Of those approached, the overall consent rate was 90% (*n* = 85) (95% CI 85%, 96%; range across Trusts 81 (*n* = 21) to 100% (*n* = 25)). Because of the challenges with random selection of potential particiapnts to approach, the recruitment strategy was revised for the post-intervention data collection phase to use convenience sampling methods and approach any eligible women for consent at their routine 12-week ultrasound scan. A total of 132 women identified in this way were consented (see Table 5) (range per Trust 25–49). Fourteen women recruited to the questionnaire data collection (four from the intervention arm and ten from the control arm) also took part in qualitative interviews.

**Table 4:** Recruitment and retention rates of pregnant women (pre-intervention) to data collection

		**Eligible**	**Randomised (denominator eligible)**		**Approached (denominator randomised)**		**Consented (denominator approached)**		**20-week questionnaire completed (denominator consented)**		**Weight measurement recorded in notes (denominator consented)**	
			***n***	**%**	***n***	**%**	***n***	**%**	***n***	**%**	***n***	**%**
**Intervention**	Trust 1 (large)	173	50	29%	26	52%	21	81%	14	67%	12	57%
	Trust 2 (small)	58	58	100%	25	43%	25	100%	14	56%	5	20%
	**Total**	**231**	**108**	**47%**	**51**	**47%**	**46**	**90%**	**28**	**61%**	**17**	**37%**
**Control**	Trust 3 (large)	242	50	21%	17	34%	14	82%	14	100%	1	7%
	Trust 4 (small)	123	41	33%	26	63%	25	96%	17	68%	11	44%
	**Total**	**365**	**91**	**25%**	**43**	**47%**	**39**	**91%**	**31**	**79%**	**12**	**31%**
**All**	**Overall total**	**596**	**199**	**33%**	**94**	**47%**	**85**	**90%**	**59**	**69%**	**29**	**34%**

**Table 5:** Recruitment and retention rates of pregnant women (post-intervention) for questionnaire data collection

		Consented	Completed baseline (12-week) questionnaire		Completed 36-week questionnaire		Completed 3-month postnatal questionnaire		Completed 6-month postnatal questionnaire		Completed 9-month postnatal questionnaire		Completed 12-month postnatal questionnaire	
		*n*	*n*	%	*n*	%	*n*	%	*n*	%	*n*	%	*n*	%
**Intervention**	NHS Trust 1 (large)	27	27	100%	10	37%	8	30%	7	30%	0	0%	9	33%
	NHS Trust 2 (small)	49	25	51%	7	14%	6	12%	5	10%	4	8%	4	8%
	**Total**	**76**	**52**	**68%**	**17**	**22%**	**14**	**18%**	**12**	**16%**	**4**	**5%**	**13**	**17%**
**Control**	NHS Trust 3 (large)	31	31	100%	14	45%	3	10%	4	13%	2	6%	1	3%
	NHS Trust 4 (small)	25	21	84%	8	32%	8	32%	6	24%	6	24%	6	24%
	**Total**	**56**	**52**	**93%**	**22**	**39%**	**11**	**20%**	**10**	**18%**	**8**	**14%**	**7**	**13%**
**All**	**Overall total**	**132**	**104**	**79%**	**39**	**30%**	**25**	**19%**	**22**	**17%**	**12**	**9%**	**20**	**15%**

Denominator in all cases is number consented

Characteristics of recruited pregnant women are summarised in Additional file 5. The maternal age and BMI of women recruited to GLOWING using both recruitment strategies were similar to the background population. In relation to deprivation, the amended recruitment strategy (i.e. convenience sample) was more reflective of the background population than the original randomisation strategy. The characteristics of recruited midwives have been reported elsewhere [36].

### Feasibility of delivering the intervention as planned

Intervention delivery was piloted in the first training day in one NHS Trust. On this occasion, one component from each of two elements (‘Weight Communication Feedback’ and ‘Weight Management Adapting Script’) was not delivered because of time constraints. Even with these omissions, the workshop was 6 h and 40 min. NH and CM reviewed and adapted intervention content to ensure feasibility of delivery in the allocated time. Thereafter, all eligible midwives received the intervention as planned (i.e. all five elements and their constituent components were delivered). However, in 23% of instances, delivery was to fewer than six midwives. Number of attendees varied from three to eight. The small NHS Trust always had four or fewer midwives present, and three sessions in the larger NHS Trust had more than six. Overall, in the larger Trust, the intervention was delivered over 8 days, in line with plans. However, in the smaller Trust, two more workshops than expected were required to accommodate midwives’ working patterns and overall workforce capacity. Completion of delivery took 15 weeks (from 05/09/2016 to 14/12/2016) in the larger Trust and 7 weeks (from 10/10/2016 to 28/11/2016) in the smaller Trust. Total delivery time could be calculated for eight GLOWING training days (where they were recorded as part of the process evaluation) and ranged from 5 h and 2 min to 6 h and 24 min, with a mean of 5 h and 48 min. The most time-consuming elements were the Weight Communication and Weight Management lectures (average durations 60 and 70 min respectively).

### Intervention acceptability and relevance to practice

All midwives in the intervention arm completed and returned their evaluation form, although not all questions were answered by all participants (see Additional files 6 and 7). Overall, more midwives rated each component of the intervention as being very or somewhat useful than not very or not at all useful. The highest scoring components, with 100% of participants rating them as being very or somewhat useful, were the introduction session, the weight communication lecture, video and group discussion, and the resources. The only components with < 90% of participants rating as very or somewhat useful were the role-play and script activities in the weight communication, weight management and consolidation sessions: 78 to 86% rated these activities as very or somewhat useful, and 14 to 22% not very or not at all useful. Qualitative analysis of the free text responses included general positive statements about the intervention (*n* = 51), stating that the training was *‘interesting’*, *‘useful’*, *‘informative’*, *‘relaxed’* and *‘enjoyable’*. There were also some general negative comments (*n* = 14) relating to the facilitators reading from a script, some elements were thought to be repetitive, and the length of the day (*‘Overall an excellent day but it is a long day’*). Midwives referred to the intervention increasing their knowledge (*n* = 24) relating to the obesity and weight management evidence-base, obesity mechanisms, stigma, communication strategies and available information. This was often discussed in the context of increasing midwives’ confidence and how it would inform routine practice:

*‘Made me reflect on my knowledge base and gave me strategies to employ when communicating with women’*

*‘I now have a deeper understanding of the topic I feel more confident in my ability to use this information in the clinical setting’*

The usefulness of GLOWING for routine practice was the most frequently coded data. Ninety responses mentioned routine practice; 56 of these were prompted responses to the question ‘What do you think will be most useful to your routine practice?’ whereas 34 were included in responses to other free text questions. Midwives referred positively to being equipped with practical strategies to change their practice, use of the resources, sensitive communication strategies and signposting women to additional support services:

*‘Will change my practice with reference to evidence-based information to provide women’*

*‘Really good resources provided to support practice’*

There was frequent reference to intervention content including the lecture, reflection activities, group discussion, interactive sessions, role-play, use of scripts, development of actions plans and provision of resources (*n* = 122). There were mixed perspectives on all components with the exception of the group discussion and resources which were overall viewed positively:

*‘Again excellent resources and group discussion’*

*‘Really helpful resources for communication’*

Qualitative data also indicated that some midwives felt that the lectures were useful *(‘Outstanding presentation. Great balance of natural discussion allowed around slides without losing direction’)* but some suggested that they could have been shorter *(*‘*Lecture useful but a lot of information relayed verbally—quite long’)* (*n* = 8). Limited data were available for reflection activities (*n* = 4) with mixed views expressed (*‘Reflective activity excellent to help digest info’; ‘This* (reflection post training day) *is more something you would do as an individual’)*. Similarly, there was mixed feedback on time devoted to developing action plans *(‘Perhaps better to write action plans at a later date when on my own and I can think about this more’, ‘Action plan has helped me to plan ahead for more challenging situations’*) (*n* = 7).

By far, the most conflicting data related to the role-play (*n* = 34) and scripted activities (*n* = 12). This mixed feedback was also reflected in the quantitative data. It was clear that some midwives did not enjoy role-play, felt that there was too much of it (three sessions) and that it was not a realistic situation. Others enjoyed the role-play, found it a good opportunity to practice having the conversation or felt that there was benefit even if they did not enjoy doing it:

*‘Too much role play, feels too forced and not how I would react in practice’*

*‘Role play a little challenging but learnt from the experience - able to reflect on practice’*

*‘Enjoyed all of this session, loved the role play!’*

### Acceptability of the trial procedures amongst pregnant women

Qualitative interviews with women recruited to GLOWING showed that they were generally accepting of the initial recruitment approach and of trial processes. Reasons given for participation included *‘helping’* others, and *‘waiting anyway’* in the scan clinic. Participation eligibility being based on their BMI was not understood by two participants, and another two stated explicitly that the offer of vouchers for questionnaire completion was the main reason for participation, though these tokens of appreciation were welcomed by all women. Discussion of the ease or otherwise of questionnaire completion focused on those administered on recruitment and in the third trimester. The initial questionnaire was perceived to be self-explanatory and very simple to fill in. Two women would have preferred the option to take this questionnaire home to complete and return at their convenience, but one reported this was not offered (despite it being part of the protocol). The third trimester questionnaire was noted to be longer but, on the whole, was also simple to complete. Of those that had some difficulty in responding, challenges were couched in terms of a lack of opportunity to provide contextual information to explain their answers, e.g. a change of diet being due to a gestational diabetes diagnosis.

### Feasibility of collecting the outcome measures required for a definitive trial

All consented midwives received a baseline questionnaire (Table 3). The overall response rate (completed or partially completed questionnaire) at baseline was 89% (*n* = 66) (95% CI 82%, 96%) (range per Trust 78–100%, Table 3), in the range of ‘excellent’ [45]. Follow-up questionnaires were sent to all consented midwives, approximately one month after the last intervention training session had been delivered. The overall response rate at follow-up was 66% (*n* = 49) (95% CI 55%, 77%), in the range of ‘acceptable’ [45]. However, this masked a marked difference between the intervention and control arms of 39% (*n* = 15) and 94% (*n* = 34) respectively. Overall, 64% (*n* = 47) (95% CI 53%, 75%) of midwives returned both baseline and follow-up questionnaires. Because of the low rate of completion of the 3-month follow-up questionnaires by midwives in the intervention arm, we abandoned plans for a second follow-up at 6-months post-intervention. Instead, we sent a short form to all but one of the seventeen midwives in the intervention arm who were lost to follow-up, asking what had prevented them from returning a follow-up questionnaire. Three indicated that they had completed and returned their questionnaires; we assumed that these were lost in the post. Five indicated that the questionnaire took too long to complete and four felt that it was too complicated. Free-text comments elaborated how workload and personal circumstances represented competing demands on their time. Amongst midwives returning a questionnaire, the levels of item non-response were low, both pre- and post-intervention. Overall levels of complete data were self-reported behaviours 98.3%, self-efficacy 99.2%, outcome expectancies 99.5%, intentions 98.8%, BAOP 99.7% and vignettes 95.4%.

In both phases of data collection from pregnant women, questionnaires were provided to all those consented to take part. In the pre-intervention phase of data collection from pregnant women, when only one questionnaire was administered (at 20-week scan), overall response rate was 69% (95% CI 60%, 79%), at the upper end of the ‘acceptable’ range [45]. In the post-intervention phase, the first questionnaire had an overall response rate of 79% (95% CI 72%, 86%), considered ‘very good’ [45]. However, for the follow-up questionnaires in the third trimester and postnatal time points, response rates fell off significantly over time, despite the provision of a £10 incentive for each questionnaire returned (Table 5). Overall rates of loss to follow-up with respect to number returning a baseline questionnaire at 12 weeks’ gestation were 63% for the third trimester questionnaire and 81% by 12 months post-partum. Feedback from the participating Trusts indicated that reasons for non-completion of follow-up questionnaires included preterm delivery, loss of the pregnancy, transfer of care from recruiting Trust to another and concerns about the woman’s welfare (e.g. domestic violence).

Of the 104 women returning a questionnaire in the post-intervention phase (with questions about their discussions with midwives at their booking appointment), 10 had their booking visit at a Trust other than the one at which they were recruited. Of these, two booked at an intervention Trust but were recruited in a control Trust and four booked at a control Trust but were recruited at an intervention Trust (between-arm contamination), and four booked at a non-GLOWING site but were recruited at a trial site (one intervention, three control). Intention to treat analysis was conducted for these participants.

Amongst pregnant women returning a completed questionnaire, levels of item non-response were low for all time points. Overall levels of complete data were 87.1% for the FFQ, 96.5% for women’s report of their midwives’ behaviours and 98.9% for the psychosocial questionnaire and the PPAQ. Recording of women’s weight in pregnancy and post-partum varied considerably across Trusts (Table 6). Across all Trusts, pregnancy weight data were available for approximately one-third of woman, with lower rates of availability for postnatal weight.

**Table 6:** Rates of availability of weight data in notes (post-intervention)

		**Pregnancy medical records (post-intervention)**		**3rd trimester weight card**		**3 months postnatal weight card**		**6 months postnatal weight card**		**9 months postnatal weight card**		**12 months postnatal weight card**	
		***n***	**%**	***n***	**%**	***n***	**%**	***n***	**%**	***n***	**%**	***n***	**%**
**Intervention**	NHS Trust 1 (large)	15	56%	9	33%	7	26%	7	26%	0	0%	9	33%
	NHS Trust 2 (small)	0	0%	7	14%	5	10%	5	10%	4	8%	4	8%
	**Total **	**15**	**20%**	**16**	**21%**	**12**	**16%**	**12**	**16%**	**4**	**5%**	**13**	**17%**
**Control**	NHS Trust 3 (large)	6	19%	13	42%	3	10%	4	13%	2	6%	1	3%
	NHS Trust 4 (small)	17	68%	8	32%	6	24%	6	24%	5	20%	6	24%
	**Total**	**23**	**41%**	**21**	**38%**	**9**	**16%**	**10**	**18%**	**7**	**13%**	**7**	**13%**
**Overall**	**Overall total**	**38**	**29%**	**37**	**28%**	**21**	**16%**	**22**	**17%**	**11**	**8%**	**20**	**15%**

Denominator in all cases is number consented (see Table 5)

## Discussion

This pilot study aimed to explore the feasibility of delivering a behaviour change intervention to support midwives’ implementation of weight management guidelines using rehearsal cluster RCT methods. The frameworks provided by Bugge et al. [46] and Shanyinde et al. [47] informed interpretation of the findings and recommendations regarding changes to the intervention and trial protocol that would be necessary before proceeding to a full-scale cluster RCT. The logistics of running a multi-centre trial [47] were assessed, albeit in only four sites within the same geographical region. In general, it seemed that ‘all components of the protocol worked together’, though with the need for some minor modifications. Bugge and colleagues [46] suggest that there are ‘…four potential options in regard to addressing the problems identified *(in a pilot trial)*: (1) adapting the intervention; (2) adjusting the clinical context within which the intervention would be delivered; (3) amending elements of the trial design; or (4) a combination of any of the former’. They propose consideration of whether identified issues (and potential solutions) are problems only in the context of a trial; might also be problematic if the intervention was to be rolled out in real life; or are problems in both contexts. For GLOWING, any issues to do with feasibility and acceptability of the intervention delivery to midwives would be problematic both in any future large-scale trial and if the intervention was to become part of routine practice. Issues relating to recruitment and retention of midwives and pregnant women for data collection would only be relevant to a future trial context. Given that issues arising from the pilot trial were primarily related to participant retention for data collection, solutions are therefore mainly related to the third option: amending elements of the trial design.

A notable success was that the intervention was delivered to all eligible midwives. In the GLOWING pilot, however, all intervention workshops were delivered by the same two members of the research team (NH and CM). This model would not be feasible or scalable to a larger national trial nor to widespread implementation of the intervention should a future trial show it to be effective and cost-effective. An alternative model, e.g. using ‘train the trainer’ methods to deliver the intervention to midwives in local areas, could overcome this. Additional workshops were needed in the small Trust, with fewer attendees than targeted. In future, opening up workshops to attendees from more than one Trust could alleviate this problem. Adherence to the intervention, and acceptability thereof, were acceptable. In particular, there was consistent positive feedback on how directly relevant the intervention content was to midwives’ routine practice. The least popular components of the intervention related to scripted role-plays. These were included as a rehearsal behaviour change technique, which is an important element of social cognitive theory. Additionally, the majority of midwives rated these activities as being useful, and some positive feedback was provided in qualitative data. Rather than removing role-play activities completely from a future trial, we suggest reducing their number. Although the script was developed with two research midwives, we also suggest working with community midwives to co-develop this further and make it more relevant to their real-world experience.

Recruitment of Trusts was not problematic, nor was their cluster randomisation to intervention or control arms. The nature of the intervention was such that blinding of intervention arm subsequent to baseline data collection was not possible. Recruitment of midwives, both to the intervention and for data collection, was highly successful, suggesting that no changes would be required for a larger-scale trial. In a cluster RCT of the GLOWING intervention, eligible pregnant women would only need to be identified and consented for data collection; while the randomisation approach was unsatisfactory, the convenience sampling approach worked well. Research midwives had access, via the women’s medical records, to almost all of the information they needed to ascertain eligibility as these data were collected at the earlier booking appointment. Although the revised approach resulted in a convenience rather than a random sample, routine scan appointments are allocated based on gestational age, determined by last menstrual period date. Moreover, we did not observe any clinically important differences when comparing the GLOWING participant characteristics with the background population. Therefore, the lack of randomisation is unlikely to be a source of systematic recruitment bias and is a usual approach in pregnancy trials. Data from qualitative interviews with participating women demonstrated the recruitment and data collection processes were acceptable, although alternative methods for completion should be offered in a larger trial. There are also a number of caveats that would need to be considered in a revised trial protocol. We did not complete a screening log in the post-intervention phase for women who were deemed ineligible or declined participation. In a definitive trial data, these data would be required to enable reporting in line with CONSORT standards and to inform conclusions regarding selection bias. During routine antenatal care, ‘cross-over’ of pregnant women between Trusts does occur, leading to potential contamination between arms. This could have a range of implications for questionnaire responses and additional inclusion criteria should be that the women had their booking appointment at the same NHS Trust that they are recruited from. While pre-intervention questionnaire data collection for midwives and baseline questionnaire completion for post-intervention women were high (89% and 79% respectively), loss to follow-up was a significant problem with questionnaire return at follow-up dropping both for intervention arm midwives (39%) and for women (ranged between 30% at 36 weeks’ gestation and 9% at 9 months postnatal). An unexpected finding was the marked differential loss to follow-up between midwives in the intervention and control arms. The reasons given for non-response were plausible, relating primarily to competing demands on the midwives’ time. However, these challenges might have been expected to affect all midwives equally. One possible explanation is that questionnaire completion was one task too many for the intervention group midwives, on top of workshop attendance, completion of the evaluation form, subsequent implementation of weight management guidelines and (for some) participating in a focus group, before being asked to complete their follow-up questionnaire. Reducing the burden of data collection would be essential in any future definitive trial, to increase retention rates and thereby reduce the risk of attrition bias. For pregnant women, the burden of data collection would be significantly reduced by administering questionnaires only at the 12-week scan appointment and in the 3rd trimester. The first of these time points is the most appropriate time to collect data on women’s perceptions of their midwife’s behaviour at the booking visit, while this is still fresh in their minds. Data on outcomes directly relevant to the NICE guidelines (i.e. diet, PA, weight) are not routinely recorded elsewhere, and should be the focus of a third trimester questionnaire. Loss to follow-up at this time point could be reduced by sending questionnaires earlier in the third trimester (in the pilot trial, one reason for non-response was delivery before 36 weeks) and by offering alternative methods of questionnaire completion (e.g. electronic). Collection, with consent, of email addresses and phone numbers could also improve retention.

The strengths of this pilot trial are that there was an explicit statement of aims and objectives, with clear focus on feasibility and acceptability outcomes, in a published protocol [34], and comprehensive data collection, using both quantitative and qualitative methods on which to judge the areas of success and refinements required prior to a definitive trial. We also used existing frameworks to interpret findings and identify solutions [46, 47]. However, there are also limitations. There were no explicit progression criteria specified in protocol and the trial was designed and commenced prior to publication of CONSORT extension for pilot trials [35], though this was mitigated by use of the relevant checklist in this report.

## Conclusions

Providing support for midwives implementation of weight management guidelines into routine practice remains a high priority and area of unmet need [48]. This pilot trial has demonstrated feasibility of delivering a theory-based behaviour change intervention to all eligible midwives. Delivery of trial procedures, including data collection (albeit with some amendments required for a definitive trial), was also feasible. Clinical midwives reported that the resources and training delivered as part of the intervention were directly relevant to, and supported, their clinical practice, resulting a strong level of acceptability of the intervention. This rigorously conducted pilot trial was delivered to all eligible midwives and has provided detailed and constructive information to inform refinement of intervention and trial protocol, enhancing confidence that a definitive trial could be carried out and informing proposed changes to intervention and to trial processes.

### Supplementary Information

**Additional file 1.** CONSORT 2010 checklist of information to include when reporting a pilot or feasibility trial*.

**Additional file 2.** Adapted NICE guideline recommended behaviours developed for the GLOWING questionnaire.

**Additional file 3.** Specific components of the intervention training day.

**Additional file 4.** Training evaluation form.

**Additional file 5.** Characteristics of recruited GLOWING women compared with the background population of women with an obese BMI in the participating Trusts.

**Additional file 6.** Questionnaire evaluation of intervention arm midwives experience of the GLOWING intervention.

**Additional file 7.** Free text responses to evaluation form.

## Data Availability

The questionnaires used in GLOWING and the anonymised quantitative data will be available on request (from corresponding author); results will be provided on condition that the required approvals are in place and clear justification as to why the data are needed and how they will be used.

## Abbreviations

BAOP: Beliefs about obese persons
BMI: Body mass index
CONSORT: Consolidated Standards of Reporting Trials
EPOC: Effective Practice and Organisation of Care
EPIC: European Prospective Investigation into Cancer
GLOWING: GestationaL Obesity Weight management: Implementation of National Guidelines
ICC: Intracluster correlation
ID: Identifier
NHS: National Health Service
NICE: National Institute for Health and Care Excellence
PA: Physical activity
PPAQ: Pregnancy Physical Activity Questionnaire
RCOG: Royal College of Obstetricians and Gynaecologists
RCT: Randomised controlled trial
SCT: Social cognitive theory
SPIRIT: Standard Protocol Items: Recommendations for Intervention Trials
TIDieR: Template for Intervention Description and Replication
UK: United Kingdom
UPBEAT: UK Pregnancy Better Eating and Activity Trial
