# Supplementary material for: The Gestational Obesity Weight Management: Implementation of National Guidelines (GLOWING) study: a pilot cluster randomised controlled trial
Source: Pilot Feasibility Stud. 2024 Mar 1;10:47. doi: 10.1186/s40814-024-01450-2 (PMC10905942; doi:10.1186/s40814-024-01450-2)
Supplement: Supplementary file 2 — Additional file 2. Adapted NICE guideline recommended behaviours developed for the GLOWING questionnaire. [file 40814_2024_1450_MOESM2_ESM.pdf]

Additional file 2: **Adapted NICE guideline recommended behaviours developed for the GLOWING questionnaire**

Behaviour Category	Adapted recommendation
**Weight Communication**	1. During the booking appointment, explain to all pregnant women why BMI is required<br>2. During the booking appointment, explain to all pregnant women how BMI will be used to plan their subsequent care<br>3. Discuss weight status (their individual BMI) with all pregnant women<br>4. Use sensitive language when discussing weight with pregnant women who have a BMI in the obese range<br>5. Address the weight-related concerns of pregnant women who have a BMI in the obese range (e.g. worries they have about weight gain)
**Risk Communication**	6. During the booking appointment, explain the pregnancy-related risks to themselves when women have a BMI in the obese range<br>7. During the booking appointment, explain the risks to their baby to women with a BMI in the obese range<br>8. During the booking appointment, explain to women with an obese BMI that they should not try to lose weight while pregnant<br>9. During the booking appointment, explain how the obesity risks will be managed by health professionals caring for them during pregnancy<br>10. During the booking appointment, explain to women with an obese BMI the risks of gaining too much weight during pregnancy
**Diet and Nutrition**	11. Discuss eating habits with all pregnant women<br>12. Ask all pregnant women if they have any concerns about their diet<br>13. Give all pregnant women practical and tailored advice about their diet<br>14. Discuss pregnancy myths about what and how much to eat during pregnancy with all pregnant women
**Physical Activity**	15. Ask all pregnant women about their levels of physical activity<br>16. Ask all pregnant women if they have any concerns about the amount of physical activity they do<br>17. Explain to all pregnant women the benefits of being physically active for their own health<br>18. Explain to all pregnant women the benefits of being physically active for their baby<br>19. Advise all women that moderate-intensity physical activity is safe to her and her baby<br>20. Advise all pregnant women of the number of minutes per day they should be active<br>21. Explain to all pregnant women that they should avoid being sedentary as much as possible<br>22. Provide practical advice for all pregnant women about how to build physical activity into their daily life
**Weight Management**	23. Advise all pregnant women that having a healthy diet and being active during pregnancy will help them to achieve a healthy weight postnatally<br>24. In the last trimester, encourage pregnant women who had a booking BMI in the obese range to lose weight after their pregnancy<br>25. Encourage women with an obese booking BMI to lose weight when you see them postnatally

	26. Reassure women that a gradual postnatal weight loss will not adversely affect their ability to breastfeed<br>27. Reassure women that a gradual postnatal weight loss will not adversely affect their quantity of breast milk<br>28. Reassure women that a gradual postnatal weight loss will not adversely affect their quality of breast milk<br>29. Discuss evidence-based gestational weight gain recommendations with women when they ask you about weight gain in pregnancy
**Referrals and Signposting**	30. Refer all women with an obese BMI to a dietitian or appropriately trained health professional for assessment and personalised advice<br>31. Advise all pregnant women to seek information or advice on diet, activity, and weight management from a reputable source<br>32. Provide details of appropriate community-based services to all women who want support to lose weight postnatally
