# Supplementary material for: The Gestational Obesity Weight Management: Implementation of National Guidelines (GLOWING) study: a pilot cluster randomised controlled trial
Source: Pilot Feasibility Stud. 2024 Mar 1;10:47. doi: 10.1186/s40814-024-01450-2 (PMC10905942; doi:10.1186/s40814-024-01450-2)
Supplement: Supplementary file 3 — Additional file 3. Specific components of the intervention training day. [file 40814_2024_1450_MOESM3_ESM.docx]

Additional file 3: Specific components of the intervention training day

| **Intervention components** | **Time allocation** |
| --- | --- |
| 1. Introduction session:  Introduction to the research  Introduction to the training session | 20 minutes |
| 2. Weight communication session:  Lecture  Video and group discussion  Reflection on practice after video  Role play/script  Group discussions and feedback after role play  Reflection on role play and adapting script | 3 hours 10 minutes with 15 minute break |
| 3. Weight management session:  Lecture  Video and group discussion  Reflection on practice after video  Role play/script  Group discussions and feedback after role play  Reflection on role play and adapting script | 2 hours 15 minutes with 15 minute break |
| 4. Consolidation session:  Timed role play  Adapting script  Making action plans | 1 hour |
| 5. Conclusions, summing up and evaluation form completion | 30 minutes |
