# Supplementary material for: The Gestational Obesity Weight Management: Implementation of National Guidelines (GLOWING) study: a pilot cluster randomised controlled trial
Source: Pilot Feasibility Stud. 2024 Mar 1;10:47. doi: 10.1186/s40814-024-01450-2 (PMC10905942; doi:10.1186/s40814-024-01450-2)
Supplement: Supplementary file 4 — Additional file 4. Training evaluation form. [file 40814_2024_1450_MOESM4_ESM.pdf]

Additional file 4: Training evaluation form

glowing

Resources	1. Very useful	2. Somewhat useful	3. Not very useful	4. Not at all useful
• Training pack				
• Information to share with pregnant women				
Any additional comments about the resources?				

Facilities and training delivery	1. Very useful	2. Somewhat useful	3. Not very useful	4. Not at all useful
• Venue location				
• Venue facilities				
• Lunch				
• Refreshments				
• Facilitator				
• Observer				
Any additional comments about the facilities and training delivery?				

**What do you think will be most useful to your routine practice?**
**Is there anything missing from GLOWING that would help with your practice?**
**Any general/additional comments about the training day?**

Thank you!!
