# Supplementary material for: The Gestational Obesity Weight Management: Implementation of National Guidelines (GLOWING) study: a pilot cluster randomised controlled trial
Source: Pilot Feasibility Stud. 2024 Mar 1;10:47. doi: 10.1186/s40814-024-01450-2 (PMC10905942; doi:10.1186/s40814-024-01450-2)
Supplement: Supplementary file 5 — Additional file 5. Characteristics of recruited GLOWING women compared with the background population of women with an obese BMI in the participating Trusts. [file 40814_2024_1450_MOESM5_ESM.pdf]

Additional file 5: Characteristics of recruited GLOWING women compared with the background population of women with an obese BMI in the participating Trusts

	1 Year Trusts Data for women with obese BMI (n=2948)	GLOWING Data		
		Sample 1: 20 weeks (n=59)	Sample 2: 12 weeks (n=104)	Total sample (n=163)
**Booking BMI, kg/m²** Mean (SD)	35.2 (4.8)	34.5 (4.0)	37.2 (6.6)	36.2 (6.0)
**Maternal age, years** Mean (SD)	29.0 (5.6)	28.1 (4.9)	29.9 (5.4)	29.2 (5.3)
**Deprivation quintile, n (%)** Q1 (most deprived)	1425 (48.4)	34 (57.6)	55 (52.9)	89 (54.6)
Q2	733 (24.9)	16 (27.1)	20 (19.2)	36 (22.1)
Q3	327 (11.1)	3 (5.1)	11 (10.6)	14 (8.6)
Q4	244 (8.3)	5 (8.5)	10 (9.6)	15 (9.2)
Q5 (least deprived)	217 (7.4)	1 (1.7)	7 (6.7)	8 (4.9)
