# Supplementary material for: The Gestational Obesity Weight Management: Implementation of National Guidelines (GLOWING) study: a pilot cluster randomised controlled trial
Source: Pilot Feasibility Stud. 2024 Mar 1;10:47. doi: 10.1186/s40814-024-01450-2 (PMC10905942; doi:10.1186/s40814-024-01450-2)
Supplement: Supplementary file 6 — Additional file 6. Questionnaire evaluation of intervention arm midwives experience of the GLOWING intervention. [file 40814_2024_1450_MOESM6_ESM.pdf]

Additional file 6: Questionnaire evaluation of intervention arm midwives experience of the GLOWING intervention

**Questionnaire components**	**Responses for each question, n**	**1. Very useful or 2. Somewhat useful, n (%)**	**3. Not very useful or 4. Not at all useful, n (%)**
1. Introduction session:			
Introduction to the research	67	67 (100%)	0
Introduction to the training session	66	66 (100%)	0
Additional comments (free text)	8	N/A	N/A
2. Weight communication session:			
Lecture	67	67 (100%)	0
Video and group discussion	66	66 (100%)	0
Reflection on practice after video	64	63 (98%)	1 (2%)
Role play/script	65	51 (78%)	14 (22%)
Group discussions and feedback after role play	64	62 (97%)	2 (3%)
Reflection on role play and adapting script	64	55 (86%)	9 (14%)
Additional comments (free text)	25	N/A	N/A
3. Weight management session:			
Lecture	67	66 (99%)	1 (1%)
Video and group discussion	66	64 (97%)	2 (3%)
Reflection on practice after video	65	62 (95%)	3 (5%)
Role play/script	63	51 (81%)	12 (19%)
Group discussions and feedback after role play	64	63 (98%)	1 (2%)
Reflection on role play and adapting script	64	52 (81%)	12 (19%)
Additional comments (free text)	25	N/A	N/A
4. Consolidation session:			
Timed role play	66	55 (83%)	11 (17%)
Adapting script	64	55 (86%)	9 (14%)
Making action plans	65	60 (92%)	5 (8%)
Additional comments (free text)	22	N/A	N/A
5. Resources:			
Training pack	66	66 (100%)	0
Information to share with pregnant women	66	66 (100%)	0
Additional comments (free text)	12	N/A	N/A
6. Facilities and training delivery:			
Venue location	65	61 (94%)	4 (6%)
Venue facilities	66	63 (95%)	3 (5%)
Lunch	66	65 (98%)	1 (2%)
Refreshments	66	66 (100%)	0
Facilitator	65	65 (100%)	0
Observer	63	63 (100%)	0
Additional comments (free text)	15	N/A	N/A
What do you think will be most useful to your routine practice? (free text)	56	N/A	N/A
Is there anything missing from GLOWING that would help with your practice? (free text)	11	N/A	N/A
Any general/additional comments about the training day? (free text)	40	N/A	N/A
