# Supplementary material for: The Gestational Obesity Weight Management: Implementation of National Guidelines (GLOWING) study: a pilot cluster randomised controlled trial
Source: Pilot Feasibility Stud. 2024 Mar 1;10:47. doi: 10.1186/s40814-024-01450-2 (PMC10905942; doi:10.1186/s40814-024-01450-2)
Supplement: Supplementary file 7 — Additional file 7. Free text responses to evaluation form. [file 40814_2024_1450_MOESM7_ESM.pdf]

Additional file 7: Free text responses to evaluation form

Questionnaire	Responses
Introduction session	1. Practical chance to use tape measure/bioimpedence<br>2. Delivered well, made interesting<br>3. Really useful information about fat adipose cells and pregnancy and breastfeeding<br>4. Shame the researchers had to follow a script<br>5. Good handouts - felt relaxed<br>6. Really useful session - new information I will use in practice<br>7. A necessary evil<br>8. Excellent introduction
Weight communication session	1. Outstanding presentation. Great balance of natural discussion allowed around slides without losing direction. Very thought provoking<br>2. Don't particularly like role play<br>3. Not so keen on role play. Packs really good.<br>4. Needed further discussion re semantics and language used. Realistic video but disappointing that this practice is the norm. Made me reflect on my knowledge based and gave me strategies to employ when communicating with women<br>5. Offered a real awareness regarding attitudes, beliefs regarding obesity. Initiated interest in what could be achieved.<br>6. All really relevant to practice<br>7. Enjoyed resources to give to women<br>8. Love the literature!<br>9. Excellent resources<br>10. Really helpful resources for communication<br>11. Watching a role play in video form and adapting scripts from there may have been more useful. Role play in forced situations very unnatural<br>12. Prefer real life case studies than role play as staged<br>13. Would have preferred to see the opposite video 'an example of good weight communication' rather than role play. I will adapt script based on individual woman rather than in general<br>14. Reflective activity excellent to help digest info<br>15. Good to do role play and discussion. Difficult to engage when reading from notes<br>16. Really enjoyed the role play. Really enjoyed the adipose cell slide - new information for me<br>17. Gives idea of how to start discussing BMI in a sensitive manner<br>18. Interesting to relate to non-verbal communication-body language<br>19. Role play was low key which was good<br>20. Helpful to discuss how to tackle the subject<br>21. Role play highlighted how balance of power can influence response. Professional to be aware of this<br>22. Really well planned and presented. Various activities.<br>23. I always find role play a bit of a false situation and find discussion (group) more helpful.<br>24. Too much role play, feels too forced and not how I would react in practice - group discussion much more useful<br>25. Not keen on role play. Adapting script - good way to reflect.

Weight management session	1. Lecture session was long with lots of info to go through. Felt [facilitator] struggled with keeping on time and went quickly through info<br>2. Brilliant session! I feel better equipped and more confident to talk to mums<br>3. Very useful practical advice - to be able to put into practice<br>4. As above. But does break up session.<br>5. Probably GP discussion and adaptation of own "script" could have been discussed without the need for role play - some really good resources and strategies that are achievable<br>6. Helpful tools to feel confident in offering women advice and support.<br>7. Again excellent resources and group discussion.<br>8. Didn't like role play session. Think most of info was covered in discussion and video. Adapting script is difficult as discussion has to be adapted to the women individually<br>9. Lecture useful but a lot of information relayed verbally -quite long. Hard to adapt script as every conversation tailored to individual.<br>10. Will adapt script to each individual woman - depending on her situation<br>11. I think the lecture could be reduced. I would prefer to look through the pack separately to session rather than skim through<br>12. Enjoyed all of this session, loved the role play!<br>13. Some info repeated from morning session<br>14. Long lecture just after lunch. Some repetition from morning session<br>15. Will change my practice with reference to evidence based information to provide women<br>16. Role play gives an idea of what to say<br>17. Good information advice on small changes to make<br>18. Role play useful but didn't like doing it<br>19. Very informative, lots of information given. I now have a deeper understanding of the topic I feel more confident in my ability to use this information in the clinical setting.<br>20. Some of the information in lecture didn't need to be all read out - just the main points would have been enough. Information on practice seems repetitive from last communication session, very repetitive role play - same as video feedback didn't think needed to do role play again and go through pack. Overall a good day but some aspects repetitive.<br>21. Very informative<br>22. As above - I always find role play a bit of a false situation and find discussion (group) more helpful<br>23. Too much time for personal reflection, thought it could have been as effective straight into group discussion.<br>24. Some information repetitive<br>25. Have made me reflect on my current practice and feel I will change my practice
Consolidation session	1. Hard to judge accurately to make this part as need longer to think about making action plans.<br>2. Action plan has helped me to plan ahead for more challenging situations<br>3. Helpful to build confidence and familiarisation with resources and advice.<br>4. Very informative - definitely change practice<br>5. Too long to make action plans. Could have just discussed it.<br>6. Found it more difficult than anticipated to introduce resource pack

	7. Group discussion and planning ahead useful<br>8. Role play not realistic<br>9. Don't like using script<br>10. Adapting script to own is more realistic<br>11. Timed role play did give an idea of how long might take but isn't necessarily realistic to actual situation<br>12. Helped me to think outside of box was useful - realised that hospital info is not enough<br>13. Good to practice how to use info leaflets<br>14. Enjoyed this - fun!<br>15. Perhaps better to write action plans at a later date when on my own and I can think about this more<br>16. Just needed time to put in my own words. Good for examples with what to say, but false setting so found it hard.<br>17. Adapting script to suit my conversational style was very useful. Seeing how long it took to deliver the information was good - lots of information which is targeted is easily conveyed<br>18. Overall a good day but some aspects repetitive. Action plans were a repetitive activity.<br>19. Don't like role play but it wasn't too painful<br>20. Happy in this group to do this. However, some may not feel comfortable with this.<br>21. Stressful, but good support<br>22. This is more something you would do as an individual (reflection post training day)
Resources	1. Excellent resource pack<br>2. Really good resources provided to support practice<br>3. Think Tommy's leaflet and classes leaflet very useful<br>4. Brilliant<br>5. Really good resources for pregnant women<br>6. Lovely, bright colours, easy to read<br>7. Fantastic resources - love the Tommy book!<br>8. Excellent resources, very informative and visual<br>9. Excellent resource pack to address with patients<br>10. Excellent resource pack<br>11. Would be great to give to all women not just those with BMI > 30<br>12. Very useful, easy to read and understand
Facilities	1. Felt it was tough for the lecturer<br>2. Would like windows! But know that's impossible.<br>3. No more role play!<br>4. Well looked after with tea/coffee and snacks. Thankyou<br>5. Toilets out of order. Chairs very uncomfortable.<br>6. Enjoyed the day<br>7. Chairs were a little uncomfortable but comfort break were great and refreshments/food was excellent. Toilet facilities (broken today)<br>8. A bit interruption from observer. Sometimes difficult as reading from script. Overall good.<br>9. Enjoyable day - thank you<br>10. Good to be onsite, good to have lots of refreshments and a nice lunch. Enjoyable session by Cath.<br>11. Excellent, interactive, extremely informative

	12. No air in room or natural light but can't change this. Long time in same room.<br>13. Education room not appropriate for whole day - no natural light - cramped<br>14. Excellent study day (even with role play)<br>15. Very relaxed approach
What do you think will be most useful to your routine practice?	1. Having increased knowledge able to share reliable info.<br>2. Gain knowledge and confidence when talking to women regarding raised BMI<br>3. Resources to give to women.<br>4. Everything<br>5. Info packs for women. Resources to use.<br>6. Chance to work out how I am going to incorporate in practice<br>7. Resource packs<br>8. I will use this in booking and follow-up<br>9. Use of resource pack<br>10. Knowledge of how to access information.<br>11. More confident about discussing exercise and diet. Happy to use resources and leaflets<br>12. Snack ideas. Cheap healthy meals. Clarifying urban myths - revisiting NICE guidelines<br>13. Confidence to initiate discussion/intervention re weight management<br>14. All of today's session will inform/support practice.<br>15. Changes to providing individualised care. Local classes.<br>16. Leaflets<br>17. Implementing this into practice<br>18. Having the resources to refer to.<br>19. All information re weight management and what we should be telling people was useful<br>20. Has given me the knowledge to confidently discuss weight management and how to deliver information i.e. different strategies<br>21. Facilities and support available in the community. Ideas about how to overcome barriers, more knowledge about current guidelines for pregnant mothers.<br>22. Extra knowledge, more in depth. Won't be worried if women want further info. I feel I'll be giving evidence based info<br>23. All of the info packs will be really useful<br>24. The Tommy's handbook - resource pack<br>25. Resource pack very useful and feel that they are going to be used by my women<br>26. Literature available - understanding<br>27. Understanding how to give information sensitively<br>28. Using the information given<br>29. Having information packs to give out<br>30. Being more aware of how to communicate with the women<br>31. Signposting to services available<br>32. Packs<br>33. Having visual aids to point out to women<br>34. Expanding my knowledge<br>35. The information in the Glowing pack<br>36. Resource booklets

	37. Feel better equipped to inform women.<br>38. Thinking about how to discuss issues. Practical resources. Problem shooting e.g. what to say if people 'don't like veg' etc.<br>39. Increase of basic knowledge<br>40. Resource pack<br>41. The resource packs and sign posts to other relevant info<br>42. Having good resource pack to share with women<br>43. The updated and in-depth knowledge and written information for women and us!<br>44. All of it<br>45. Glowing Pack<br>46. The resource pack<br>47. Resource packs - helpful when discussing weight management. Good prompts - helpful for explanations to the women.<br>48. Information packs to give women and tools to help women making small changes<br>49. Resources<br>50. Having more confidence to broach weight and weight management. Having more knowledge around subjects. Being aware of resources.<br>51. Resource packs for women my information booklet<br>52. The information packs and resource links<br>53. Prompt of leaflet pack<br>54. The information to give to women<br>55. Booklet will be useful for information for providing the women with a good insight into BMI and Healthy Eating in Pregnancy<br>56. Handing out Glowing packs
Is there anything missing from GLOWING that would help with your practice?	1. Really useful training session. Very good resource pack. Very beneficial in day to day role.<br>2. Probably need a 5 minute break out of the room.<br>3. I felt there was too much role play and reading from script was not real.<br>4. A good day with lots of useful info<br>5. At the moment nothing<br>6. More practical resources<br>7. No. Already packed in enough into one day.<br>8. Quite small room so noisy when doing role play but understand that this is unavoidable<br>9. There are lots of things that I will use in my practice.<br>10. Nothing I can think of<br>11. It would be nice if all pregnant women could be issued with packs.
Any general/additional comments about the training day?	1. Thanks<br>2. Really enjoyed it. Works well being in small group.<br>3. Enjoyed the training.<br>4. Great day!<br>5. Interesting day<br>6. Very good and useful for practice - thank you<br>7. Brilliant! Thanks, I feel more equipped<br>8. Very enjoyable, informative and relative to practice<br>9. Very good day and helpful resources. It think it was a bit too long.<br>10. Role play a little challenging but learnt from the experience - able to reflect on practice.

	11. Really good. I have increased my knowledge. I feel more confident about information and advice to women<br>12. Great day - thank you<br>13. Really helpful. Will directly affect my practice.<br>14. Good day - enjoyable and informative<br>15. Really interesting - if no role play/videos, day wouldn't need to be as long<br>16. Long day. Too much read from slides (not spontaneous)<br>17. Very useful day to use in practice.<br>18. Good use of resources. Brilliant that we have been shown where to access valuable resources<br>19. Am sure it will assist me in providing more support/advice/care to the women - especially those who are overweight<br>20. Overall an excellent day but it is a long day - feel consolidation wasn't necessary although I can see how it could help some people. Definitely did not need to hear other people's action plans.<br>21. Everyone was very friendly - info very useful, will be great to use in practice.<br>22. Thank you for lunch<br>23. Excellent training day - thank you<br>24. Really useful day. Enjoyed it!<br>25. Good session and good to be in small groups.<br>26. Enjoyed the day<br>27. Excellent day overall.<br>28. Well thought out training<br>29. It was great<br>30. Thank you both good day<br>31. Brilliant day - thank you<br>32. Didn't really enjoy role play - makes me self-conscious and therefore didn't get anything from it (quite funny though)<br>33. Good, informal atmosphere, small groups encourage discussion. Realistic aims/outcomes<br>34. Didn't feel any benefit from the audio - could just be read. The day could have been more condensed<br>35. Better and more informative than I was expecting<br>36. Didn't enjoy role play<br>37. Useful training that has had a positive impact on my future practice.<br>38. Didn't feel script/role play achieved much. Perhaps better to discuss what is done already and how, with a prompt leaflet, this could be extended<br>39. It will make the BKG appt longer so I will have to adapt my appointment to make time for this.<br>40. I don't feel the role play was useful at all. Felt parts of session were a little condescending. I don't feel session needs to be full day.
